# Pediatric Obesity: Diagnostic and Therapeutic Approaches in the Context of International Guidelines With a Focus on Polish Practice

**DOI:** 10.7759/cureus.101545

**Published:** 2026-01-14

**Authors:** Paweł M Łuczak, Jakub Perediatkiewicz, Paweł Liszka, Konrad Puchalski, Klaudia M Patrzykąt, Klaudia M Olejnik-Chlewicka, Wojciech Urbański, Marta Zasiadła, Jakub Brodowski, Agata Ogórek

**Affiliations:** 1 Pediatrics, Independent Public Healthcare Complex – Hospital in Iłża, Iłża, POL; 2 Gastroenterology, New Hospital in Olkusz, Olkusz, POL; 3 Endocrinology, Jan Mikulicz-Radecki University Clinical Hospital, Wrocław, POL; 4 Psychiatry, Voivodeship Specialist Hospital in Wrocław, Wrocław, POL; 5 Internal Medicine, 109 Military Hospital in Szczecin, Szczecin, POL; 6 Nephrology, Voivodeship Integrated Hospital in Kielce, Kielce, POL; 7 Internal Medicine, Jan Mikulicz-Radecki University Clinical Hospital, Wrocław, POL; 8 Cardiology, Tadeusz Sokołowski University Clinical Hospital No. 1 Pomeranian Medical University (PUM) in Szczecin, Szczecin, POL; 9 Neurology, Jan Mikulicz-Radecki University Clinical Hospital, Wrocław, POL; 10 Oncology, Lower Silesian Center of Oncology, Pulmonology, and Hematology, Wrocław, POL

**Keywords:** bariatric surgery, childhood obesity, clinical practice guidelines, glp-1 receptor agonist, pediatric obesity, screening

## Abstract

Pediatric obesity is a common chronic disease with serious long-term health consequences. This narrative review compares current international and national recommendations for the diagnosis and management of pediatric obesity. We focus on guidance from the American Academy of Pediatrics (AAP), the World Health Organization (WHO), the UK National Institute for Health and Care Excellence (NICE), and the Polish pediatric position statement, with reference to other relevant European and expert consensus documents. We also summarize key pathophysiological mechanisms relevant to clinical assessment, diagnostic work-up, and treatment selection. We highlight areas of consensus and clinically important differences, especially in diagnostic thresholds, risk stratification, and screening for obesity-related comorbidities. Across frameworks, family-based lifestyle and behavioral intervention remain fundamental components of care. Pharmacotherapy, particularly glucagon-like peptide-1 (GLP-1) receptor agonists such as liraglutide and semaglutide, and metabolic/bariatric surgery are recommended as adjunctive options for carefully selected adolescents with severe obesity and significant comorbidities. In Poland, published reports and policy analyses suggest that implementation is often constrained by delayed access to specialist obesity services and structured lifestyle-behavioral support, as well as limited reimbursement for anti-obesity medications. As a result, escalation of care may occur late, even among adolescents who meet guideline-based criteria.

## Introduction and background

Childhood obesity is a prevalent chronic disease and a major public-health concern, as it frequently tracks into adulthood and is associated with early-onset obesity-related complications and comorbidities [1,2]. International Classification of Diseases, Eleventh Revision (ICD-11) classifies obesity as a chronic, complex disease, and clinicians still rely on BMI-for-age to screen and stage severity [3]. However, recent expert consensus suggests that BMI should be primarily treated as a screening tool, and diagnosis should reflect adiposity-related dysfunction rather than body size alone [4].

The global burden remains high: in 2022, obesity affected about 159 million children and adolescents aged five to 19 years worldwide, and prevalence continues to rise [5,6]. In Poland, the WHO European Regional Obesity Report 2022 reports that in 2016, the age-standardized prevalence of obesity was 12.5% among school-aged children aged five to nine years (boys 16.8%, girls 8.0%) and 7.2% among adolescents aged 10-19 years (boys 10.4%, girls 3.8%). In both age groups, obesity was more common in boys than in girls [7].

**Figure 1:**
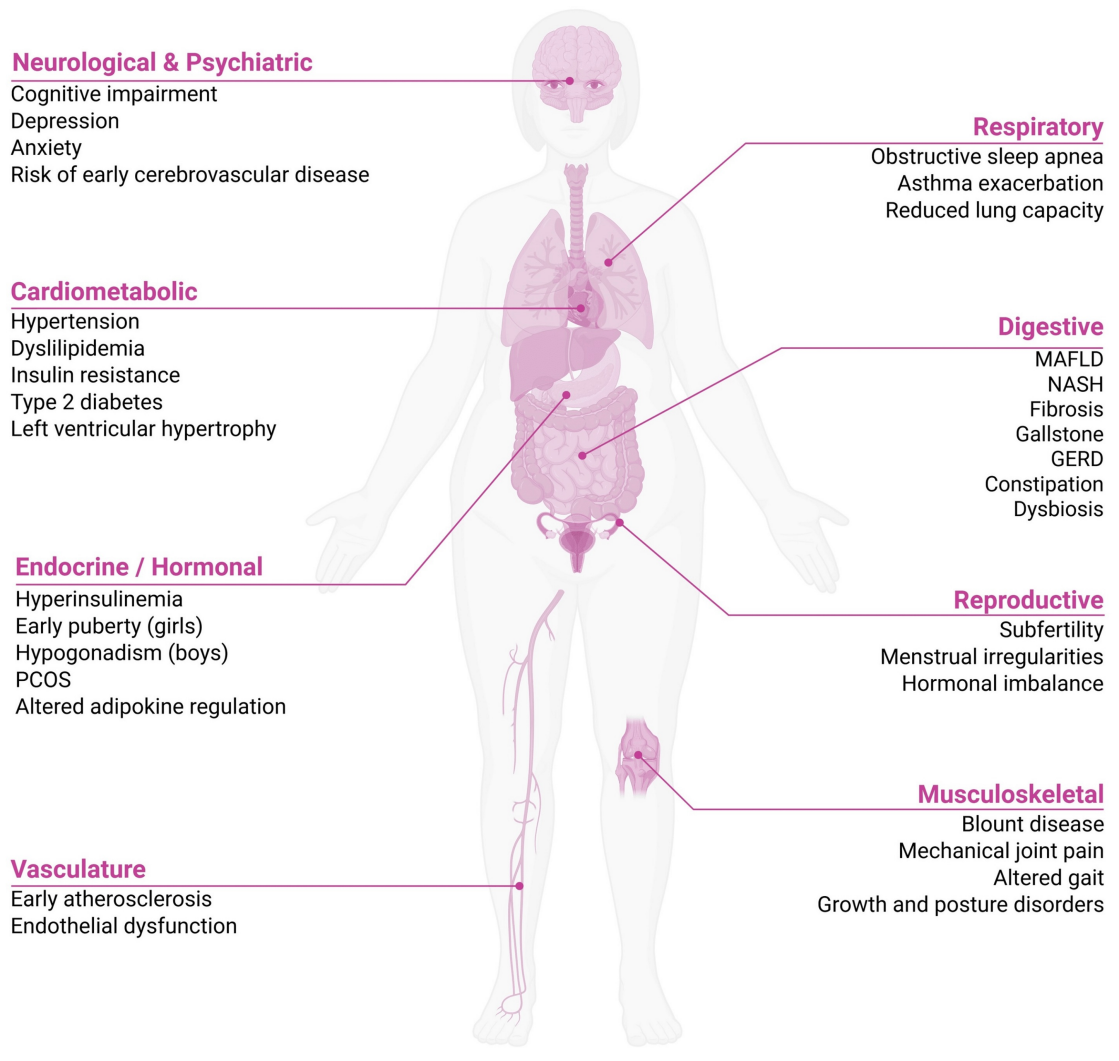
Health impact of pediatric obesity across organ systems Schematic representation of the major obesity-related complications in children and adolescents, affecting neurological and psychiatric, cardiometabolic, endocrine/hormonal, vascular, respiratory, digestive, reproductive, and musculoskeletal systems. MAFLD: metabolic-associated fatty liver disease; NASH: non-alcoholic steatohepatitis; GERD: gastro-esophageal reflux disease; PCOS: polycystic ovary syndrome This image is original to this study and was created by the authors using BioRender.com (BioRender, Toronto, Canada).

In this narrative review, we compare guidance on pediatric simple obesity primarily from the American Academy of Pediatrics (AAP), World Health Organization (WHO)/WHO Europe, National Institute for Health and Care Excellence (NICE), and the Polish pediatric position statement, with reference to other relevant expert consensus documents where applicable [7-12]. We highlight differences in diagnostic thresholds and staging. We compare screening for comorbidities and criteria for treatment escalation. We summarize treatment options: family-based behavioral therapy, pharmacotherapy (especially glucagon-like peptide-1 (GLP‑1) receptor agonists), and metabolic bariatric surgery [8-10,12-14]. Figure 1 summarizes the health impact of pediatric obesity across organ systems.

## Review

Methods

We conducted a narrative review to compare contemporary recommendations for the diagnosis and management of pediatric simple obesity across major international and national guidance documents. As this is a narrative, guideline-focused review, Preferred Reporting Items for Systematic Reviews and Meta-Analyses (PRISMA) reporting was not applied. We did not perform a formal risk-of-bias assessment or a fully reproducible study-selection workflow typical of systematic reviews.

On 1 November 2025, we searched PubMed/Medical Literature Analysis and Retrieval System Online (MEDLINE), Google Scholar, and the Cochrane Library for English- and Polish-language publications from January 1, 2015, to November 1, 2025. We also searched guideline repositories and official websites of key organizations (AAP, WHO/WHO Europe, NICE, and Polish pediatric guidance) and consulted other relevant expert consensus documents, including the Endocrine Society and European Association for the Study of Obesity (EASO) resources on dietary management. We screened reference lists of key guidelines and reviews to identify additional sources.

We used combinations of Medical Subject Headings (MeSH) where applicable, and free-text terms related to pediatric obesity and guidance documents. Key terms included "child OR adolescent," "obesity OR overweight," and "guideline OR consensus OR position statement," combined with topic-specific terms such as "BMI percentile," "comorbidity," "pathophysiology," "screening," "GLP-1," "liraglutide," "semaglutide," "metabolic/bariatric surgery," "implementation gaps," and "limitations."

We included clinical practice guidelines and consensus statements as the primary evidence base. We used systematic reviews and randomized trials to contextualize and support specific guideline statements where applicable, without implying uniform effectiveness across all settings. This review synthesizes published sources; no original data were collected or analyzed. We excluded adult-only studies and case reports. We extracted key elements from each document, including definitions, diagnostic thresholds, comorbidity screening recommendations, and management strategies. When recommendations differed across documents, we prioritized the most recent, pediatric-specific guidance and higher-authority society guidelines, and explicitly noted key discrepancies. We synthesized areas of concordance and highlighted clinically relevant differences.

Pathophysiology of obesity

The pathophysiology of childhood obesity is multifactorial and reflects the interaction of biological, environmental, and psychosocial determinants [2,10,15]. Key processes include a chronic imbalance between energy intake and expenditure, dysregulation of central hunger and satiety pathways, altered neuroendocrine control of appetite, adipose tissue dysfunction, and individual genetic susceptibility [1,2]. Understanding these mechanisms is essential for accurate diagnosis and for selecting appropriate therapeutic interventions, which we discuss in the following sections.

Energy Balance and Neuroendocrine Regulation

Body-weight regulation depends on the hypothalamus-gut-adipose axis, which integrates neural and hormonal signals within the arcuate nucleus of the hypothalamus. Appetite is governed by two key neuronal pathways: the anorexigenic proopiomelanocortin/cocaine- and amphetamine-regulated transcript pathway (POMC/CART) and the orexigenic neuropeptide Y/agouti-related peptide pathway (NPY/AgRP). The POMC/CART pathway acts mainly through melanocortin signaling (including α-MSH binding to MC4R). In contrast, the NPY/AgRP pathway increases food intake and suppresses anorexigenic signaling, in part by inhibiting POMC neurons via GABAergic transmission [1]. These circuits are further modulated by peripheral hormones such as leptin, ghrelin, and insulin. Leptin and postprandial insulin help reduce appetite and support higher energy expenditure. Ghrelin has the opposite effect: it rises during fasting and signals the initiation of a meal. Incretins (GLP-1 and GIP) additionally promote satiety and support insulin secretion, and orexins (hypocretins) link appetite regulation with arousal and energy expenditure [1,15].

In children exposed to an obesogenic environment characterized by highly palatable, energy-dense foods, insufficient sleep, and low physical activity, these regulatory mechanisms can become dysregulated [1,2]. This dysregulation is associated with leptin and insulin resistance, altered ghrelin dynamics after meals, and reduced responsiveness to satiety signals such as GLP-1. Highly palatable foods also activate dopaminergic reward pathways and promote hedonic eating, which can override homeostatic appetite control and reinforce overeating. Over time, these changes support a persistent positive energy balance and contribute to the development and maintenance of pediatric obesity [16].

Adipose Tissue Dysfunction and Metabolic Consequences

Adipose tissue exists in several biologically distinct forms. White adipose tissue primarily stores energy, whereas brown adipose tissue is specialized for thermogenesis. Beige adipocytes represent an inducible intermediate phenotype with features of both white and brown fat [1,2]. In pediatric obesity, expansion of white adipose tissue occurs through a combination of adipocyte hyperplasia and hypertrophy. Hypertrophic adipocytes are particularly prone to hypoxia because vascular supply cannot keep pace with tissue growth. This microenvironment promotes oxidative stress and adipocyte dysfunction, shifting adipokine secretion toward a pro-inflammatory profile characterized by increased leptin, TNF-α, and IL-6 and reduced adiponectin. Immune-cell infiltration follows, most notably polarization of macrophages from an anti-inflammatory M2 phenotype toward a pro-inflammatory M1 state, driving low-grade chronic inflammation [1]. Over time, adipose tissue becomes fibrotic, and its capacity to safely store lipids declines. This promotes progressive insulin resistance, impaired lipid handling, and ectopic lipid accumulation in the liver, skeletal muscle, and pancreas, accelerating cardiometabolic complications [1].

The metabolic impact of adiposity depends strongly on fat distribution. Excess visceral adipose tissue is closely linked to dyslipidemia, non-alcoholic fatty liver disease, and early atherosclerotic changes, whereas subcutaneous depots may have a neutral or partially protective metabolic profile [1,17].

Genetic and Epigenetic Determinants

Genetic predisposition substantially modulates individual susceptibility to weight gain, and heritability estimates for childhood BMI range from approximately 40% to 70% in twin and family studies. In most patients, obesity has a polygenic basis, resulting from the cumulative effect of many common variants. Each variant has a small impact on appetite regulation, energy expenditure, and fat distribution. Genome-wide association studies have identified numerous loci associated with increased obesity risk, with variants near the *FTO* and *MC4R* genes and other genes involved in hypothalamic signaling showing some of the strongest associations. These polymorphisms typically promote higher energy intake (e.g., preference for calorie-dense foods and impaired satiety) and do not cause a primary defect in basal metabolic rate [1,18].

In a small subset of children, obesity results from single gene defects affecting key components of the leptin-melanocortin pathway. Monogenic forms most commonly involve mutations in *MC4R, LEP, LEPR, POMC, PCSK1, SH2B1, MRAP2*, or *ADCY3*, leading to early-onset, severe obesity with marked hyperphagia and, in some cases, additional endocrinological abnormalities [17,18]. Syndromic forms of obesity (e.g., Prader-Willi or Bardet-Biedl syndrome) combine excessive weight gain with dysmorphic features, neurodevelopmental delay, and multisystem involvement. Although these conditions are rare, they are clinically important because they require targeted diagnostic evaluation and, in selected cases, specific therapies [1,18].

Beyond DNA sequence variation, growing evidence points to a role for epigenetic mechanisms. These include DNA methylation, histone modifications, and non-coding RNAs. In the future, they may help assess long-term obesity risk [19]. Adverse intrauterine and early postnatal exposures (including maternal obesity, gestational diabetes, intrauterine growth restriction, smoking during pregnancy, exposure to endocrine-disrupting chemicals, and overnutrition in early life) may modify epigenetic regulation of genes involved in appetite control, insulin signaling, and adipogenesis [2,17,19]. These epigenetic changes may persist, disrupting energy homeostasis and metabolic function across the course of life and predisposing to obesity and cardiometabolic disease [19].

Environmental, Behavioral, and Psychosocial Factors

Although genetic and epigenetic factors shape susceptibility to obesity, the rise in childhood obesity is largely driven by environmental and lifestyle changes. Contemporary societies are characterized by an obesogenic environment, meaning that children and adolescents live in settings that facilitate a chronic positive energy balance through easy access to highly palatable, energy-dense foods and reduced opportunities for physical activity [2,7,13]. Dietary patterns in many children and adolescents with obesity are dominated by processed foods high in simple sugars and saturated fats, with low intake of vegetables, fruits, whole grains, and dietary fiber. Frequent consumption of sugar-sweetened beverages, large portion sizes, and snacking between meals further increases daily energy intake. Additional environmental factors associated with obesity include shorter breastfeeding duration, feeding with high-protein infant formula, and early introduction of complementary foods [2,7]. Another important contributor is a sedentary lifestyle, characterized by increasing screen time and reduced physical activity among children and adolescents. These behaviors lower total energy expenditure and adversely affect cardiorespiratory fitness. Chronic sleep restriction, common in school-aged children and adolescents, further promotes weight gain through changes in ghrelin, leptin, and cortisol secretion and through reduced impulse control related to food choices [1,11,16].

Diagnostic criteria and evaluation

BMI-Based Definitions and Classification

To diagnose obesity in children, clinicians should confirm excess adiposity, determine disease severity, and assess for possible secondary causes of obesity as well as early obesity-related comorbidities. Currently, the main parameter used to assess pediatric patients is BMI. BMI should be used as a screening tool in children and adolescents and interpreted using sex- and age-specific reference standards according to the selected growth-chart framework (percentiles or z-scores) [8-10,12]. Because cut-off values differ across international and national systems, eligibility for investigations and treatment may vary between countries. According to the AAP, NICE, and the Polish position statement, BMI-based thresholds are used to define overweight, obesity, and severe obesity to identify children at the highest risk of complications [8,9,12]. Measures of central adiposity (waist circumference or waist-to-height ratio) may refine cardiometabolic risk assessment, but they are not applied consistently across all guidelines [4,20]. Table 1 summarizes the key differences between the AAP, NICE, and Polish recommendations. 

**Table 1:** Comparison of diagnostic definitions and screening recommendations for pediatric overweight and obesity across guidance documents This table is an author‑created synthesis based on the cited guidance documents. NICE NG246 is a life‑course guideline and does not provide a prescriptive pediatric laboratory algorithm; comorbidity testing is guided by clinical judgement and existing child‑specific standards. Fatty liver terminology varies across documents (e.g., NAFLD/MAFLD). ABPM: ambulatory blood pressure monitoring; ALT: alanine aminotransferase; AAP: American Academy of Pediatrics; BMI: body mass index; BP: blood pressure; CDC: Centers for Disease Control and Prevention; GI: gastrointestinal; HbA1c: glycated hemoglobin; HDL‑C: high‑density lipoprotein cholesterol; LDL‑C: low‑density lipoprotein cholesterol; MAFLD: metabolic dysfunction‑associated fatty liver disease; MRI: magnetic resonance imaging; NICE: National Institute for Health and Care Excellence; non‑HDL‑C: non–high‑density lipoprotein cholesterol; OGTT: oral glucose tolerance test; RCPCH: Royal College of Paediatrics and Child Health; SD: standard deviation; TC: total cholesterol; TG: triglycerides; ULN: upper limit of normal; UK: United Kingdom; WC: waist circumference; WHO: World Health Organization; WHtR: waist‑to‑height ratio Sources: AAP 2023 [8], NICE NG246 [10], Mazur et al., 2022 [9].

Domain	AAP 2023	NICE (NG246)	Poland (Mazur et al., 2022)
Population covered	Children and adolescents 2–18 years; full clinical practice guideline covering evaluation and treatment of overweight and obesity.	Children and young people ≥2 years; life‑course obesity guideline with limited pediatric‑specific diagnostic and laboratory algorithms.	Infants, children, and adolescents 0–18 years; separate recommendations for <5 years and ≥3 years.
Primary anthropometric tool and charts	BMI‑for‑age percentile using CDC growth charts (with severe‑obesity extensions).	BMI centile on UK‑WHO/RCPCH growth charts; interpretation based on age- and sex‑adjusted centiles/z‑scores.	<5 years: weight‑for‑length/height z‑score (WHO). ≥3–18 years: BMI percentile, preferably Polish OLA/OLAF charts; WHO 5–19 charts acceptable if national references are unavailable.
Overweight/obesity thresholds	Overweight: BMI ≥85th to <95th percentile. Obesity: BMI ≥95th percentile.	Overweight: BMI ≥91st centile (≈ +1.34 SD). Clinical obesity: BMI ≥98th centile (≈ +2.05 SD).	<5 years: overweight 2–3 SD; obesity >3 SD above WHO median. ≥3–18 years: overweight >85th percentile (>+1 SD), obesity >97th percentile (>+2 SD) in national BMI charts.
Severe obesity definition	Severe obesity: Class 2—BMI ≥120% of the 95th percentile or BMI ≥35 kg/m² (whichever is lower). Class 3—BMI ≥140% of the 95th percentile or BMI ≥40 kg/m² (whichever is lower).	BMI ≥99.6th centile (≈ +2.68 SD).	BMI >3 SD (≈99.9th centile) in children >5 years; associated with very high cardiometabolic risk.
Central adiposity (waist indices)	BMI is the primary measure; routine waist circumference is not required but may be used as an optional risk marker.	Consider measuring waist circumference and calculating the waist‑to‑height ratio (WHtR) to estimate central adiposity. WHtR: 0.40–0.49 (healthy), 0.50–0.59 (increased risk), ≥0.60 (high risk).	Recommends measuring waist circumference. Up to 16 years: WC >90th percentile (OLA/OLAF) suggests abdominal obesity; in older adolescents, adult cut‑offs may be used (94 cm boys, 80 cm girls).
Blood pressure	Measure blood pressure at every visit from age ≥3 years in children with overweight/obesity; evaluate abnormal values per pediatric hypertension guidelines.	Blood pressure assessment is part of comorbidity evaluation; use clinical judgment for further work‑up after initial assessment, particularly if BMI ≥98th centile.	Blood pressure measurement is recommended in all children with overweight/obesity; use ABPM or home BP when white‑coat/masked hypertension is suspected; if hypertension is confirmed, assess target‑organ damage.
Glucose metabolism	Evaluate for dysglycaemia in children ≥10 years with obesity. Consider evaluation in ages 10–18 years with overweight if risk factors are present. Tests: fasting plasma glucose, OGTT, or HbA1c.	No prescriptive pediatric laboratory algorithm; use clinical judgment to assess comorbidities after initial evaluation, especially if BMI ≥98th centile.	Fasting plasma glucose recommended in all children ≥6 years with overweight/obesity; repeat every 2–3 years (earlier if risk increases). OGTT (1.75 g/kg, max 75 g) if fasting glucose is abnormal or risk is high. HbA1c should not be used as the sole screening test.
Lipid profile	Fasting lipid panel in all children ≥10 years with overweight or obesity; may be considered in ages 2–9 years with obesity.	No prescriptive pediatric laboratory algorithm; use clinical judgment to assess comorbidities after initial evaluation, especially if BMI ≥98th centile.	Fasting lipid profile (TC, LDL‑C, HDL‑C, TG) recommended in all children with overweight/obesity from age 2 years; if normal—repeat every 2 years; if abnormal—repeat every 6 months during treatment. Emphasis on non‑HDL‑C and triglycerides.
Liver disease screening (fatty liver)	In children ≥10 years with obesity: screen with ALT; consider ALT in children ≥10 years with overweight if risk factors are present. Further imaging/hepatology work‑up guided by ALT values and clinical context.	No prescriptive pediatric laboratory algorithm; use clinical judgment to assess comorbidities after initial evaluation, especially if BMI ≥98th centile.	Fatty liver disease is highlighted as a frequent GI complication. First‑line: liver enzymes and abdominal ultrasound; ALT >2× ULN considered clinically relevant. If fibrosis is suspected: elastography or MRI and noninvasive fibrosis scores; liver biopsy reserved for selected cases.

Clinical Assessment and Red Flags

In the evaluation of a child with obesity, a detailed clinical history and physical examination are essential to identify contributing factors and obesity-related complications. The history should include dietary habits, physical activity level, sedentary time, sleep patterns, and psychosocial factors (including family dynamics and mental health) that may influence body weight [8-10,12]. Clinicians should also look for clues suggesting a secondary cause of obesity, such as medication use, symptoms of endocrine disorders, or neurodevelopmental delay, indicating an underlying condition [8,9]. The physical examination should go beyond anthropometric measurements and assess key elements, including blood pressure and features of dysmorphism or developmental abnormalities suggestive of genetic disorders. It should also evaluate for signs of comorbidities (e.g., acanthosis nigricans indicating insulin resistance, hepatomegaly suggesting fatty liver disease, or orthopedic abnormalities) [1,2,8,9]. Because children with obesity may experience disturbances in linear growth and pubertal development, recording height and assessing pubertal stage are also recommended [8-10].

Laboratory Screening and Additional Testing

Laboratory testing should match the child’s age, severity of obesity, and clinical findings. Its main aim is early detection of cardiometabolic complications and selective exclusion of secondary causes [8-10]. The 2023 AAP guideline presents a step-by-step approach to metabolic screening, typically starting around age 10 (earlier in higher-risk children), and recommends endocrine tests only when the clinical picture suggests an underlying disorder [8]. The Polish 2022 consensus provides similarly detailed, practice-oriented indications for laboratory tests and imaging, including targeted abdominal ultrasound for suspected fatty liver disease and sleep assessment when obstructive sleep apnea is suspected [9]. In contrast, the NICE guideline NG246 emphasizes individual risk assessment and clinical judgement and does not propose a single fixed pediatric laboratory panel [12]. Overall, frameworks agree on screening for metabolic risk and comorbidities, but they differ in timing, scope, and the level of procedural detail [8-10,12].

Management of pediatric obesity

Lifestyle and Behavioral Interventions

Current international guidelines: Share a consistent position wherein lifestyle change and behavioral intervention are the first-line treatment for children and adolescents with obesity. They also define obesity as a relapsing chronic disease that requires long-term, family-centered, multidisciplinary care rather than short-term weight-loss efforts [8,9,11,12]. The main aim is to sustain health improvement. This includes metabolic stabilization, prevention or delay of comorbidities, and better psychosocial functioning, not weight loss alone [8-10].

Intensive health, behavior, and lifestyle treatment: The 2023 AAP guideline operationalizes this approach as intensive health behavior and lifestyle treatment (IHBLT). IHBLT is a structured, family-based program that delivers at least 26 hours of face-to-face contact over three to 12 months [8]. A multidisciplinary team provides it and focuses on nutrition, physical activity, and behavioral skills [17]. The AAP guideline recommends intensive, family-based behavioral treatment and, consistent with evidence from randomized trials (including family-based treatment implemented in pediatric primary care), indicates that greater treatment intensity (more contact hours) is associated with improved adiposity outcomes; however, effects vary across programs and settings and are not uniform [8,21]. European guidance, and the Polish consensus, also place lifestyle change as first-line therapy and recommend cognitive behavioral therapy (CBT) as part of behavioral management, but it does not specify clear timeframes or detailed parameters for CBT delivery [7,9,12].

Dietary, physical-activity, and behavioral components: Core components are similar across frameworks. Nutritional counseling targets an age-appropriate energy deficit while supporting normal growth. Guidelines promote higher intake of vegetables, fruits, whole grains, and fiber. They also advise limiting sugar-sweetened beverages, energy-dense snacks, and highly processed food, with attention to portion size and added sugars [7-9]. Physical activity targets align with the WHO 2020 recommendation: at least 60 minutes per day of moderate-to-vigorous physical activity (MVPA), with reduced sedentary time and recreational screen use [13]. Guidelines recommend goal setting, self-monitoring (food and activity logs), stimulus control, problem solving, and motivational interviewing [8,9,12].

Implementation and contextual factors: Implementation remains the main weakness. The AAP explicitly links treatment intensity to obesity severity and comorbidity burden and recommends referral to IHBLT when available [8]. In many European countries, especially in Central and Eastern Europe, like Poland, health systems lack enough multidisciplinary clinics, dietitians, and pediatric mental-health support to deliver intensive programs at scale [22-24]. As a result, primary care often provides brief advice instead of structured treatment [24]. This approach limits treatment effects and leads to substantial delays in referring children and adolescents with severe obesity for appropriate care [22,24]. Several reports describe this system constraint as a major barrier to better outcomes in the region [22,24,25].

Psychosocial and family determinants: Guidelines also highlight psychosocial and family context. Children with obesity often experience weight-related teasing, stigma, social isolation, and low self-esteem, which can undermine treatment engagement and mental health [4]. Family-based care is central, especially in younger children. It focuses on improving the home food environment, modelling healthy behaviors, and identifying triggers for episodes of uncontrolled eating [8,9,12]. In adolescents, programs should support autonomy, focus on personal goals and preferences, and address body image. They should match the teen’s developmental level and ability to learn new skills [8]. Active caregiver involvement in treatment increases the chances of successful lifestyle and behavior change and, in turn, supports healthier weight outcomes [8,22,25]. Schools and communities can support treatment by creating healthier food environments, strengthening physical education, and making it easier for children to walk or bike (for example, to school) [7-9,12]. Overall, lifestyle and behavioral intervention remain the cornerstone of pediatric obesity management [8-10,12]. Table 2 provides a concise comparison of key study findings across lifestyle-based interventions, pharmacotherapy, and metabolic-bariatric surgery in children and adolescents with obesity [21,26-33].

**Table 2:** Key study findings comparing interventions for pediatric obesity BMI: body mass index; BMI-SDS: BMI standard deviation score; CI: confidence interval; MD: mean difference; MBS: metabolic/bariatric surgery; pp: percentage points; RCT: randomized controlled trial; RYGB: Roux-en-Y gastric bypass; VSG: vertical sleeve gastrectomy; WC: waist circumference; IHBLT: intensive health behavior and lifestyle treatment Adiposity outcomes are reported as presented in the original studies (no conversions across metrics). BMI z-score/BMI-SDS are age- and sex-standardized measures; negative values indicate reductions in the reported adiposity measure, based on data reported in the cited primary studies and systematic reviews.

Study (year)	Intervention class	Design	Population	Intervention/comparator	Duration/intensity	Main adiposity outcome (as reported)
Franco et al., 2025 [26]	Lifestyle / multimodal behaviour-change interventions	Cochrane systematic review (33 RCTs with 5949 participants)	Adolescents (10-19y) living with obesity	Multimodal behaviour-change interventions (diet/PA/behavioural; healthcare- or community-based) vs control/usual care	Outcomes mainly at 12-18 months (some at 24 months)	BMI z-score (MD): healthcare-based -0.11 at 12-18 months; community-based -0.07 at 12 months
Franco et al.,2025 [27]	Lifestyle / multimodal behaviour-change interventions	Cochrane systematic review (34 RCTs; 6849 participants)	Children <10y living with obesity (+ parents/caregivers)	Multimodal behaviour-change interventions vs control/usual care	Outcomes mainly at 12 and 24 months	BMI z-score (MD): -0.15 at 12 months; -0.08 at 24 months (healthcare-based)
Epstein et al., 2023 [21]	IHBLT / family-based multicomponent behavioral treatment (primary care)	Randomized clinical trial (US primary care; n=452 child-parent dyads)	Children 6-12 years with overweight/obesity + parents	Family-based behavioral treatment delivered in primary care vs usual care	24 months; goal 26 sessions (individualized based on progress)	Between-group difference at 24 months in % above median BMI: -6.21 percentage points (95% CI -10.14 to -2.29)
Men et al., 2025 [28]	Exercise-only interventions	Systematic review + meta-analysis (113 RCTs; n=8514)	Children/adolescents with overweight/obesity	Exercise interventions vs control (various modalities)	Author-defined “effective dose”: ≥3 sessions/week, ≥50 min/session, ≥12 weeks	BMI (MD): -1.16 kg/m²; WC (MD): -2.29 cm; body fat % (MD): -2.07%
Torbahn et al., 2024 [29]	Pharmacotherapy (adjunct to lifestyle/behavioural intervention)	Systematic review + meta-analyses (35 RCTs; N=4331)	Children/adolescents (8.8-16.3y) with obesity	Anti-obesity medications + behaviour change vs comparator	Follow-up 6-24 months	BMI (MD): -1.71 kg/m² (95% CI -2.27 to -1.14); semaglutide BMI (MD): -5.88 kg/m² (N=201); BMI% of 95th percentile: -11.88 pp (N=668)
Kelly et al., 2020 [30]	Pharmacotherapy (adjunct to lifestyle/behavioural intervention)	Randomized, double-blind, placebo-controlled trial (n=251 randomized, 125 liraglutide; 126 placebo)	Adolescents 12-<18y with obesity and poor response to lifestyle therapy	Liraglutide 3.0 mg once daily + lifestyle vs placebo + lifestyle	56 weeks treatment (+ off-treatment follow-up)	BMI-SDS (difference vs placebo): -0.22; ≥5% BMI reduction: 43.3% vs 18.7%; ≥10%: 26.1% vs 8.1%
Weghuber et al., 2022 [31]	Pharmacotherapy (adjunct to lifestyle/behavioural intervention)	Randomized, double-blind, placebo-controlled trial (n=201; 2:1 randomization)	Adolescents 12-<18y with obesity	Semaglutide 2.4 mg once weekly + lifestyle vs placebo + lifestyle	68 weeks (BMI also assessed at week 75)	Mean % change in BMI to week 68: -16.1% vs +0.6%; estimated difference -16.7 pp (95% CI -20.3 to -13.2)
Järvholm et al., 2023 [32]	Metabolic/bariatric surgery	Randomized controlled trial (open-label, multicentre; n=50; 25 vs 25)	Adolescents 13-16y with severe obesity (mean baseline BMI 42.6 kg/m²)	Metabolic/bariatric surgery (mostly RYGB; some VSG) vs intensive non-surgical treatment (incl. initial 8-week low-calorie diet)	2-year follow-up	BMI change at 2y: -12.6 kg/m² (MBS) vs -0.2 kg/m² (non-surgical); MD -12.4 kg/m² (95% CI -15.5 to -9.3)
Ryder et al., 2024 [33]	Metabolic/bariatric surgery	Prospective multicenter observational cohort (RYGB n=161; VSG n=99; mean age 17y)	Adolescents 13-19y undergoing bariatric surgery	Bariatric surgery (RYGB or VSG); no contemporaneous non-surgical control	10-year follow-up (83% completed 10-year postoperative visits)	BMI % change at 10y: -20.0% (95% CI -22.9 to -17.1); similar for RYGB -20.6% and VSG -19.2%

Pharmacotherapy

Pharmacotherapy should be used only as an adjunct to structured nutritional counselling and lifestyle modification, rather than as a stand-alone treatment. In current guidelines, anti-obesity medications are generally reserved for adolescents with obesity who fail to achieve adequate improvement despite intensive health behavior and lifestyle interventions and for those who already present with significant obesity-related comorbidities [8,22,34].

GLP-1 receptor agonists: Among available drugs, GLP-1 receptor agonists are currently the most effective pharmacological options approved for adolescents with obesity [8,9,29]. Liraglutide 3.0 mg once daily is approved for weight management in adolescents ≥12 years with obesity and is explicitly recommended in the guideline as an adjunct to intensive lifestyle treatment in those who do not achieve sufficient response to behavioral interventions alone [8,9]. Randomized controlled trials in adolescents have shown clinically meaningful reductions in BMI and improvements in cardiometabolic risk factors with liraglutide when added to lifestyle programs [29,30]. Semaglutide 2.4 mg once weekly has subsequently been approved for weight management in adolescents ≥12 years with obesity (BMI ≥95th percentile and body weight >60 kg). Adolescent trial data indicate even greater average BMI reductions and favorable effects on cardiometabolic profiles compared with earlier agents [29,31]. However, the AAP guideline acknowledges semaglutide but provides less operational guidance than for liraglutide; most European and national pediatric documents mention semaglutide briefly, reflecting limited implementation experience and heterogeneous access [8,9]. In Central Europe, including Poland, the practical uptake of GLP-1-based pharmacotherapy in adolescents remains low despite formal approvals [24]. Liraglutide is registered for the treatment of obesity in patients aged ≥12 years [35]. In Poland, its use is not reimbursed for this indication and is restricted to clinicians experienced in pharmacotherapy of obesity who are fully aware of the potential adverse effects and monitoring requirements [9,36]. Semaglutide is also available, but it is not reimbursed for adolescent obesity [36]. In practice, its use in youth is limited to selected specialist centers and clinicians experienced in its use [24]. As a result, although guidelines increasingly recognize GLP-1 receptor agonists as effective adjunctive therapy for severe adolescent obesity, most affected children in Poland still have no real access to these medications in routine care [9,24,36].

Orlistat and metformin: Older pharmacological options like orlistat and metformin play a minor role in current treatment [29]. Orlistat, a gastrointestinal lipase inhibitor approved for use in individuals 12 years of age, produces only modest weight loss and is associated with frequent gastrointestinal adverse effects such as steatorrhea and fecal urgency, which markedly limit adherence [8,9,12]. Contemporary pediatric obesity guidelines generally regard orlistat as a low-priority option, to be considered only when newer agents are unavailable or contraindicated [8,9]. Metformin, although widely used in children and adolescents with impaired glucose tolerance, type 2 diabetes, or polycystic ovary syndrome (PCOS), is not formally licensed as an anti-obesity drug [8-10]. The Polish consensus does not recommend metformin solely to support weight loss in children and adolescents with overweight or obesity. It allows metformin mainly when there is a clear metabolic indication, such as obesity linked to a kinase suppressor (*KSR2*) gene mutation, metabolic syndrome, type 2 diabetes, or, in selected cases, PCOS with metabolic abnormalities [9]. Overall, the evidence does not support metformin as an effective weight-loss drug in pediatric obesity, and any BMI reduction is usually modest [8,9,29].

Targeted and investigational therapies: Targeted pharmacotherapy is available for a very small group of patients with monogenic obesity. Setmelanotide, an MC4R-pathway agonist, is indicated in the EU for children and adults with genetically confirmed biallelic *POMC* (including *PCSK1*) or *LEPR* deficiency and for Bardet-Biedl syndrome [1,8,9]. It can produce substantial weight loss and reduce hyperphagia in these ultra-rare conditions but has no role in common polygenic obesity and is accessible only in highly specialized centers, usually through exceptional reimbursement or research programs [8,9]. Tirzepatide, a dual GIP/GLP-1 receptor agonist, is approved in adults for obesity and type 2 diabetes. In children and adolescents with type 2 diabetes, a phase 3 trial has shown improvements in glycemic control and BMI, but tirzepatide has no pediatric obesity indication. Obesity-specific adolescent trials are ongoing, so it remains investigational in pediatric obesity [37].

Bariatric Surgery

Metabolic-bariatric surgery (MBS) offers the largest and most durable weight reduction in adolescents with severe, treatment-refractory obesity. Clinicians should consider surgery only for carefully selected patients and only in experienced multidisciplinary centers that provide long-term follow-up [8,9,12,14]. Surgery does not replace lifestyle treatment. It complements it when the medical risk is high and non-surgical treatment fails to produce meaningful improvement [8].

Eligibility and referral: Most guidance uses similar clinical thresholds. Surgery may be considered in adolescents with severe obesity (commonly BMI around ≥40 kg/m², or ≥35 kg/m² with at least one serious obesity-related comorbidity). High-risk comorbidities typically include type 2 diabetes, moderate-to-severe obstructive sleep apnea (OSA), severe hypertension, or clinically significant fatty liver disease [8-10,14]. Importantly, pubertal stage and skeletal maturity should not act as an absolute contraindication [9,14]. The team should base eligibility on medical risk, growth and development, psychosocial readiness, the ability to adhere to postoperative recommendations, and strong family support [8,9,12,14].

Preoperative assessment and prerequisites: The team should confirm the diagnosis, quantify the comorbidity burden, and exclude secondary causes when indicated. It should also assess mental health, eating patterns, substance use, and home support [8,9,14]. The Polish position statement generally recommends at least 12 months of structured conservative management before qualification [9], while the AAP stresses timely referral for surgical evaluation in eligible adolescents rather than delaying treatment until adulthood [8]. In practice, clinicians should document intensive lifestyle treatment and consider pharmacotherapy when appropriate before surgery.

Contraindications and safeguards: Surgery is not appropriate when an adolescent cannot participate in follow-up or when uncontrolled conditions would compromise safety or adherence. Typical contraindications include untreated severe psychiatric illness, substance abuse problems, inability to provide informed assent/consent, and persistent non-adherence that would predict loss to follow-up [9,14]. The team should confirm before surgery that the patient understands and accepts the need for lifelong micronutrient supplementation and follow-up and set clear expectations for the postoperative diet, supplements, and visits [14].

Procedures, outcomes, and monitoring: The most common procedures in adolescents are laparoscopic sleeve gastrectomy (SG) and Roux-en-Y gastric bypass (RYGB) [14]. Both can produce large and sustained BMI reductions and often improve major comorbidities, particularly glycemic control and OSA severity, with meaningful gains in quality of life [1]. However, surgery carries risks of perioperative complications and later nutritional deficiencies [11]. Patients need lifelong micronutrient supplementation, periodic laboratory monitoring, and a structured transition to adult obesity care [8-10].

Implementation gap: Even when guidelines support surgery for selected adolescents, access remains limited in Polish settings due to restricted availability of specialized centers, long waiting times, and uneven referral pathways. This gap contributes to the underuse of surgery among eligible adolescents despite high medical need [22,25].

## Conclusions

Childhood obesity is a chronic, multifactorial condition with important health and psychosocial consequences. Effective care relies on early recognition, a structured clinical assessment, and sustained, family-centered behavioral and lifestyle support delivered in a non-stigmatizing manner, with ongoing follow-up focused on overall health and functioning.

For some adolescents, additional treatment options may be appropriate within a multidisciplinary model of care. If treatment beyond lifestyle modification is being considered, the decision should be individualized and made in collaboration with the patient and family. Careful safety monitoring and long-term support are essential to help sustain benefits over time.
